# Incidence, risk factors, and predictive modeling of stoma site incisional hernia after enterostomy closure: a multicenter retrospective cohort study

**DOI:** 10.1186/s12876-023-02805-4

**Published:** 2023-06-10

**Authors:** Yonghuan Mao, Ling Xi, Chen Lu, Ji Miao, Qiang Li, Xiaofei Shen, Chunzhao Yu

**Affiliations:** 1 Department of General Surgery, Nanjing Drum Tower Hospital Clinical College of Nanjing Medical University, Nanjing, China; 2 Department of General Surgery, the Second Affiliated Hospital of Nanjing Medical University, Nanjing, China; 3 Department of Gerontology, Geriatric Hospital of Nanjing Medical University, Nanjing, China; 4 Department of General Surgery, Sir Run Run Hospital of Nanjing Medical University, Nanjing, China

**Keywords:** Enterostomy closure, Stoma site incisional hernia (SSIH), Risk factors, Prediction model

## Abstract

**Purpose:**

Stoma site incisional hernia (SSIH) is a common complication, but its incidence and risk factors are not well known. The objective of this study is to explore the incidence and risk factors of SSIH and build a predictive model.

**Methods:**

We performed a multicenter retrospective analysis on the patients who underwent enterostomy closure from January 2018 to August 2020. Patient's general condition, perioperative, intraoperative, and follow-up information was collected. The patients were divided into control group (no occurrence) and observation group (occurrence) according to whether SSIH occurred. Univariate and multivariate analysis were used to evaluate the risk factors of SSIH, following which we constructed a nomogram for SSIH prediction.

**Results:**

One hundred fifty-six patients were enrolled in the study. The incidence of SSIH was 24.4% (38 cases), of which 14 were treated with hernia mesh repair, and the others were treated with conservative treatment. Univariate and multivariate analysis showed that age ≥ 68 years (OR 1.045, 95% CI 1.002 ~ 1.089, *P* = 0.038), colostomy (OR 2.913, 95% CI 1.035 ~ 8.202, *P* = 0.043), BMI ≥ 25 kg/m2 (OR 1.181, 95% CI 1.010 ~ 1.382, *P* = 0.037), malignant tumor (OR 4.838, 95% CI 1.508 ~ 15.517, *P* = 0.008) and emergency surgery (OR 5.327, 95% CI 1.996 ~ 14.434, *P* = 0.001) are the independent risk factors for SSIH.

**Conclusions:**

Based on the results, a predictive model for the occurrence of SSIH was constructed to screen high-risk groups of SSIH. For patients at high risk for SSIH, how to deal with the follow-up and prevent the occurrence of SSIH is worth further exploration.

## Introduction

In recent years, the incidence of colorectal cancer in China continuously increase, seriously threatening the health of Chinese people [1]. At present, due to the improvement of surgical techniques and treatment options, the probability of low rectal cancer (the distance from tumor to the anus is 3 ~ 5 cm) being able to preserve the anus is increasing [2]. However, such patients are at high risk of anastomotic leakage, and therefore, prophylactic enterostomy has become a preferred clinical treatment option. Besides, prophylactic enterostomy is also applied in patients with intestinal perforation, ileus, inflammatory bowel disease, etc.

Enterostomy closure is usually performed after prophylactic enterostomy and it is generally considered to be a simple operation with a low incidence of serious postoperative complications. However, serious local contamination of the stoma, high suture tension, various underlying diseases or other factors can result in poor incision healing after stoma closure, which may be risk factors for stoma site incisional hernia (SSIH). It has been reported [3, 4] that the incidence of SSIH (about 35%) is higher than the incidence of incisional hernia (about 10%) after other abdominal surgeries, and further statistics are needed to determine in China. Patients with SSIH seek medical consultation mainly for repeatedly emerging abdominal mass, with or without abdominal pain, which impairs their quality of life. Although some of them can relieve after conservative treatment, some can gradually aggravate and need surgical treatment, which greatly increased the suffering of patients [5, 6]. For patients with symptoms like intestinal obstruction, bloody stools and severe abdominal pain, their condition is serious and life-threatening, and require emergency surgical treatment, such as intestinal adhesion release, hernia mesh repair, or even necrotic bowel resection, which significantly increases difficulty and risk of surgery [7]. Therefore, building a predictive model for SSIH and reducing incidence is of great significance. In this study, we reviewed the patients receiving enterostomy closure and analyzed the difference between patients suffering from SSIH or not. Based on the results, we established a prediction model for the occurrence of SSIH.

## Materials and methods

### Basic information

The patients with enterostomy closure who were treated in the Department of General Surgery, the Second Affiliated Hospital of Nanjing Medical University, Nanjing Drum Tower Hospital Clinical College of Nanjing Medical University, and Sir Run Run Hospital of Nanjing Medical University from January 2018 to August 2020 and received routine follow-up were reviewed and analyzed. All the operations and post-operative treatments that patients received were performed as routine patient care. All patients received oral laxatives combined with anal enema before enterostomy closure to ensure adequate intestinal preparation. The clinical data, surgery-related indicators and occurrence of SSIH were collected. Inclusion criteria: (1) ≥ 18 years old who underwent prophylactic enterostomy due to illness; (2) no serious underlying diseases and can tolerate general anesthesia; (3) good compliance: the patient can cooperate with the doctor's physical examination, follow-up diagnosis and treatment, so that the doctors can better obtain the data of the patient. Exclusion criteria: (1) incapacitated; (2) advanced malignant tumor; (3) tumor recurrence, death or loss during follow-up; (4) Hartmann reversal; (5) other reasons not suitable for inclusion. The patients without SSIH were selected as the control group (118 cases), and those with SSIH were selected as the observation group (38 cases).

According to the Helsinki Declaration, this study was registered at ResearchRegistry.com (the research registration unique identifying number was researchregistry8503, https://www.researchregistry.com/browse-the-registry#home/registrationdetails/637c8e812a77100021d0bd2b/). This is a retrospective cohort study. This study was approved by the institutional research ethics committee of the corresponding center. All procedures performed in our study were in line with the STROCSS criteria [8].

### Operation

**Fig. 1:**
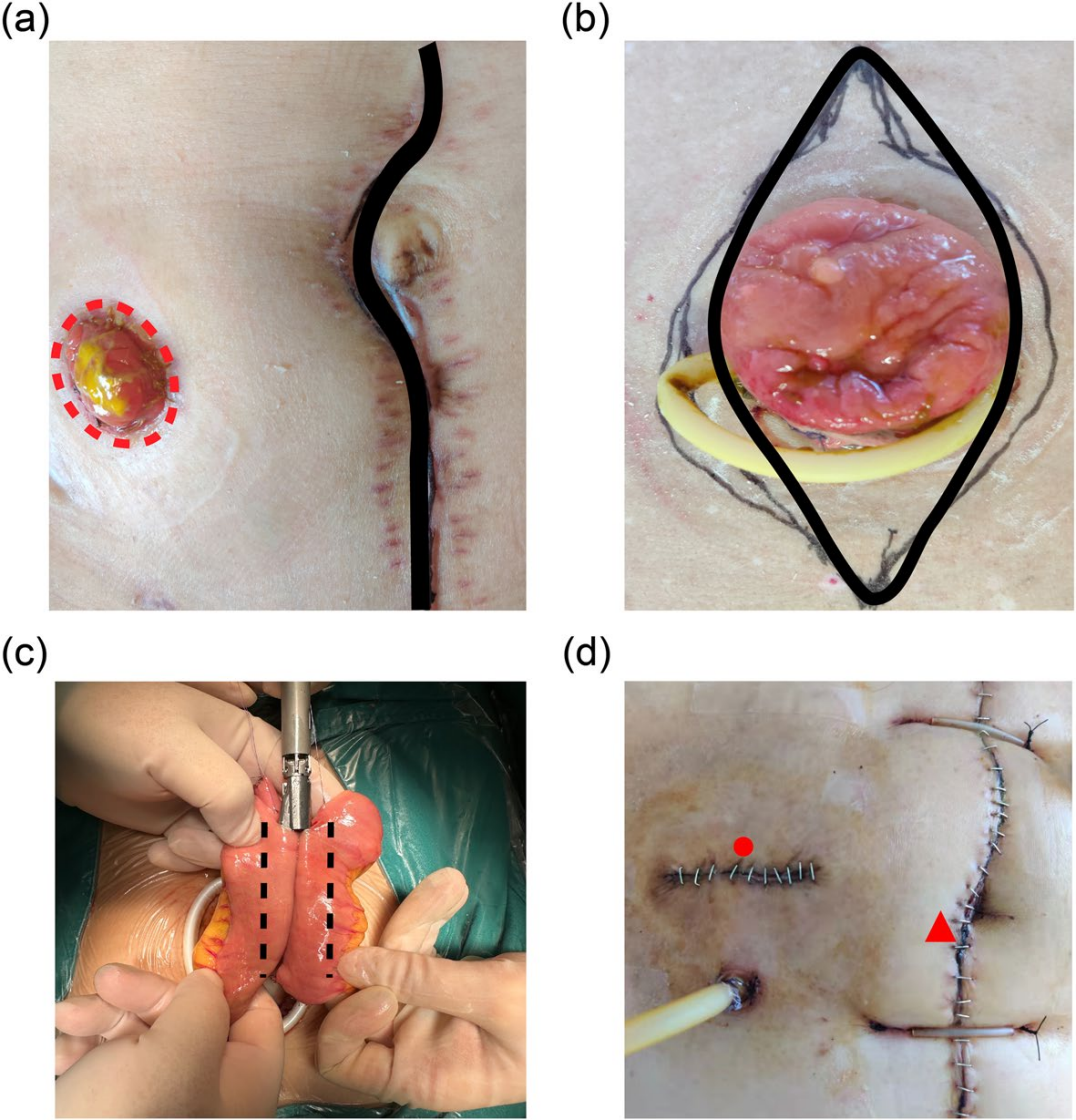
**a**: a midline incision for end stoma; **b**: a stoma incision for loop stoma; **c**: anastomosis were mechanically sutured; **d**: postoperative of stoma closure; ●: original stoma site, hernia in this area is called stoma site incisional hernia (SSIH); ▲: midline incision, hernia in this area is called incisional hernia

Enterostomy closure: The operation was performed by experienced gastrointestinal surgeons. For an end stoma, operation is performed through a midline incision (Fig. 1a), while for a loop stoma, operation is performed through stoma incision (Fig. 1b). After opening abdomen, the proximal and distal intestinal tubes of the stoma were mobilized with sufficient length. Then the intestinal tube of the original stoma was resected, folloewd by side-to-side anastomosis, or end-to-side anastomosis. All the anastomosis were mechanically sutured and strengthened by interrupted suture with the absorption line (Fig. 1c), and the fascia defect was closed by continuous suture also with the absorption line. Then, a latex tube was placed in the pelvic cavity and the other latex tube was placed behind the anastomosis. The abdomen was closed after checking for no active intra-abdominal bleeding. None of the patients had prophylactic mesh placement at the time of primary surgery or enterostomy closure.

### Postoperative treatment

The second-generation cephalosporin was used to prevent infection for 48 h after the operation. If patients presented infection-associated symptoms or signs like fever, incision redness and swelling, elevated inflammatory indicators and turbidity of drainage fluid (evaluation by the surgeon, digestive fluid, odor or fecal residue was found in the drainage fluid, and the drainage fluid was kept for culture), the anti-infection treatment would be upgraded to the third-generation cephalosporin or even higher-grade antibiotics. Antibiotics were further adjusted according to the culture results of drainage fluid. The abdominal binder was locally immobilized on abdomen for 3 months. The patients were re-examined 6 months after the operation. All patients have undergone physical examination to evaluate the occurrence of SSIH. The patient was examined in both standing and lying positions. Then the patient was told to make a forceful cough, and the surgeon placed one hand over the closed stoma site. The examiner recorded if the patient had either a palpable fascial defect with or without protrusion of abdominal contents or a global weakness around the stoma scar, without palpable fascial defect. If present or cannot be ruled out, abdominal computed tomography (CT) should be added to confirm the diagnosis. (Fig. 1d: postoperative of stoma closure; ●: original stoma site, hernia in this area is called *SSIH*; ▲: midline incision, hernia in this area is called *incisional hernia*.)

### Observation indicator

The following data of patients were collected: gender, age, body mass index (BMI), hypertension, diabetes, history of hernia repair, smoking, constipation, chronic obstructive pulmonary disease(COPD), corticosteroids, disease type (benign or malignant), stoma site (ileum or colon), stoma surgery method (open or endoscopic), stoma timing (emergency or elective), stoma method (end or loop), operation time, intraoperative bleeding, postoperative hospital stay, incision infection, incision size, incision location (midline or non-midline), follow-up time, preoperative white blood cell count(pre-WBC), preoperative neutrophil count (pre-N), preoperative lymphocyte count (pre-LY), preoperative hemoglobin (pre-Hb), preoperative c-reactive protein (pre-CRP), preoperative OPNI (prognostic nutritional index: OPNI = albumin value g/L + 5*lymphocyte count 10^9L, pre-OPNI), preoperative neutrophil/lymphocyte ratio (pre-NLR), preoperative platelet count/lymphocyte ratio (pre-PLR), postoperative white blood cell count (post-WBC), postoperative neutrophil count (post-N), postoperative lymphocyte count (post-LY), postoperative hemoglobin (post-Hb), postoperative C-reactive protein (post-CRP), postoperative OPNI (post-OPNI), postoperative NLR (post-NLR), postoperative PLR (post-PLR), and whether SSIH occurred. The preoperative routine blood test and blood biochemical test were performed before the surgery, and the postoperative routine blood test and blood biochemical test were performed 24 h after the operation.

### Statistical analysis

SPSS 23.0 statistical software was used for statistical analysis and description. The normally distributed measurement data was presented as mean ± standard deviation (m ± s), and the count data was presented by case (%). Univariate analysis was performed using χ2 test, and multivariate analysis was performed using Logistic regression analysis. *P* < 0.05 indicates that the difference is statistically significant.

### Establishment, validation, and calibration curve drawing of nomogram prediction model

The independent risk factors were introduced into the rms package of R software (R 4.0.3), the clinical nomogram prediction model was constructed, the receiver operating characteristic (ROC) curve was drawn, and the area under curve (AUC) was calculated. AUC > 0.75 was considered to have good predictive ability of the prediction model. To verify the consistency of the nomogram prediction model, a calibration curve was drawn between the predicted complication probability of the nomogram model and the actual complication probability. The C-index was used to evaluate the discrimination of the prediction model. Internal validation was performed using the Bootstrap method, with 1000 replicates from the original data, and the C-index index was corrected.

## Results

### Comparison of characteristics between the two groups

There were 156 patients undergoing enterostomy closure, including 118 in the control group (no SSIH occurrence) and 38 (SSIH occurrence) in the observation group. The incidence of SSIH was 24.4% (38/156). The results of univariate analysis showed that there were significant differences in age, BMI, stoma site, disease type, stoma timing, incision infection, pre-WBC, and post-LY between the two groups (*P*<0.05). There was no significant difference in other data between the two groups (*P*>0.05). (Table 1).

**Table 1:** Characteristics of the two groups of patients

Characteristics	control group	observation group	χ2/t value	*P* value
Cases	118(75.6%)	38(24.4%)		
Age	60.7 ± 13.0	67.5 ± 11.4	3.139	0.002 ^a^
Gender			1.184	0.277
Male	79	29		
Female	39	9		
BMI(kg/m^2^)	23.3 ± 2.9	25.0 ± 3.0	3.139	0.002 ^a^
Stoma site			6.873	0.009 ^a^
Ileum	53	8		
Colon	65	30		
Stoma surgery method			1.940	0.164
Open	53	22		
Endoscopic	65	16		
Stoma method			3.453	0.063
End	51	23		
Loop	67	15		
Disease type			4.650	0.031 ^a^
Benign	44	7		
Malignant	74	31		
Stoma timing			8.329	0.004 ^a^
Emergency	46	25		
Elective	72	13		
Hypertension			0.110	0.740
Y	40	14		
N	78	24		
Diabetes			0.372	0.542
Y	17	4		
N	101	34		
Smoking			0.641	0.423
Y	21	9		
N	97	29		
History of hernia repair			0.273	0.601
Y	4	2		
N	114	36		
Constipation			0.001	0.976
Y	3	1		
N	115	37		
COPD			0.071	0.79
Y	5	2		
N	113	36		
Corticosteroids			0.024	0.878
Y	7	2		
N	111	36		
Incision infection			5.853	0.016 ^a^
Y	5	6		
N	113	32		
Incision size(cm)	17.2 ± 3.7	16.6 ± 3.5	0.855	0.397
Incision location			0.147	0.702
Midline	57	17		
Non-midline	61	21		
Follow-up time (months)	35.6 ± 15.2	33.9 ± 15.6	0.585	0.562
Operation time(minutes)	180.8 ± 74.4	185.7 ± 80.3	0.346	0.73
Blood loss(ml)	114.4 ± 140.3	119.7 ± 155.3	0.2	0.842
Post-hospital stay(days)	10.8 ± 4.9	11.9 ± 6.9	1.07	0.286
Pre-WBC(*10^9/L)	5.8 ± 1.7	6.4 ± 2.2	2.655	0.009 ^a^
Pre-N(*10^9/L)	3.3 ± 1.4	4.0 ± 2.3	1.821	0.075
Pre-LY(*10^9/L)	1.7 ± 0.6	1.8 ± 0.7	0.919	0.36
Pre-Hb(g/L)	132.3 ± 19.0	132.4 ± 17.0	0.031	0.975
Pre-CRP(mg/L)	4.3 ± 7.2	4.6 ± 5.5	0.293	0.77
Pre-OPNI	48.3 ± 5.5	49.1 ± 6.0	0.743	0.459
Pre-NLR	2.3 ± 3.0	3.2 ± 4.2	1.243	0.221
Pre-PLR	132.6 ± 71.4	153.1 ± 125.3	0.962	0.341
Post-WBC(*10^9/L)	9.1 ± 3.2	9.5 ± 3.5	0.677	0.5
Post-N(*10^9/L)	7.4 ± 3.3	10.0 ± 12.0	1.331	0.191
Post-LY(*10^9/L)	1.0 ± 0.6	1.3 ± 1.1	2.043	0.043 ^a^
Post-Hb(g/L)	122.0 ± 19.7	119.1 ± 24.4	-0.743	0.458
Post-CRP(mg/L)	40.4 ± 32.7	36.0 ± 39.4	-0.694	0.489
Post-OPNI	40.8 ± 4.8	41.0 ± 6.9	0.224	0.823
Post-NLR	10.4 ± 8.3	9.2 ± 6.4	-0.864	0.389
Post-PLR	230.3 ± 153.7	214.5 ± 116.0	-0.581	0.562

*BMI* Body mass index, *Y* Yes, *N* No, *COPD* Chronic obstructive pulmonary disease, *post* Postoperative, *pre* Preoperative, *WBC* White blood cell count, *N* Neutrophil count, *LY* Lymphocyte count, *Hb* Hemoglobin, *CRP* C-reactive protein, OPNI = albumin value g/L + 5*lymphocyte count 10^9L, NLR = neutrophil/lymphocyte ratio, PLR = platelet count/lymphocyte

^a^ *P* < 0.05

### Logistic multivariate regression analysis was performed on the indicators significantly correlated with the occurrence of SSIH in the univariate outcome analysis

**Fig. 2:**
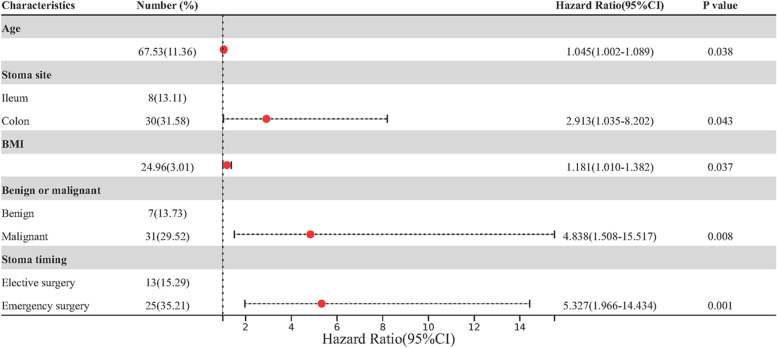
Forest plot

Based on the results of univariate analysis, we further performed multivariate analysis to find out the independent risk factors for SSIH. The results showed that age ≥ 68 years (OR 1.045, 95% CI 1.002 ~ 1.089, *P* = 0.038), colostomy (OR 2.913, 95% CI 1.035 ~ 8.202, *P* = 0.043), BMI ≥ 25 kg/m^2^ (OR 1.181, 95% CI 1.010 ~ 1.382, *P* = 0.037), malignancy (OR 4.838, 95% CI 1.508 ~ 15.517, *P* = 0.008), and emergency surgery (OR 5.327, 95% CI 1.996 ~ 14.434, *P* = 0.001) were independent risk factors for SSIH (Table 2). The forest plot based on the results of univariate analysis is shown in Fig. 2. Subgroup analysis of loop stoma and end stoma show that malignant tumor is a high risk factor for end stoma to SSIH, age and stoma timing (emergency/elective surgery) are high risk factors for loop type stoma to SSIH (Table 3).

**Table 2:** SSIH multivariate analysis

Characteristics	Regression coefficient	Standard error	Wald value	odds ratio(95%CI)	*P* value
Age	0.044	0.021	4.316	1.045(1.002–1.089)	0.038 ^**b**^
Stoma site	1.069	0.528	4.099	2.913(1.035–8.202)	0.043 ^**b**^
BMI	0.167	0.080	4.339	1.181(1.010–1.382)	0.037 ^**b**^
Benign or malignant	1.576	0.595	7.028	4.838(1.508–15.517)	0.008 ^**b**^
Stoma timing	1.673	0.509	10.818	5.327(1.966–14.434)	0.001 ^**b**^
incision infection	0.667	0.806	0.684	1.948(0.401–9.455)	0.408
Preoperative WBC	0.224	0.120	3.520	1.251(0.990–1.582)	0.061
Postoperative LY	0.156	0.253	0.379	1.169(0.712–1.919)	0.538

*WBC* White blood cell count, *LY* Lymphocyte count

^**b**^ *P* < 0.05

**Table 3:** SSIH subgroup multivariate analysis of loop-stoma and end-stoma

Characteristics	Regression coefficient	Standard error	Wald value	odds ratio(95%CI)	*P* value
*End stoma*					
BMI	0.162	0.118	1.866	1.176(0.932–1.483)	0.172
Benign or malignant	2.103	0.702	8.215	7.482(1.890–29.629)	0.004 ^c^
Preoperative WBC	0.344	0.497	0.479	1.410(0.533–3.732)	0.489
Preoperative N	0.170	0.541	0.098	1.185(0.410–3.423)	0.754
Postoperative CRP	-0.006	0.010	0.321	0.994(0.975–1.014)	0.571
*Loop stoma*					
Age	0.155	0.044	12.514	1.168(1.072–1.273)	< 0.001 ^d^
BMI	0.132	0.110	1.432	1.141(0.919–1.417)	0.231
Stoma timing	2.331	0.885	6.933	10.285(1.814–58.301)	0.008 ^d^

*BMI* Body mass index, *WBC* White blood cell count, *N* Neutrophil count, *CRP* C-reactive protein

^**c**^ *P* < 0.05

^d^ *P* < 0.05

### Building a nomogram clinical prediction model

**Fig. 3:**
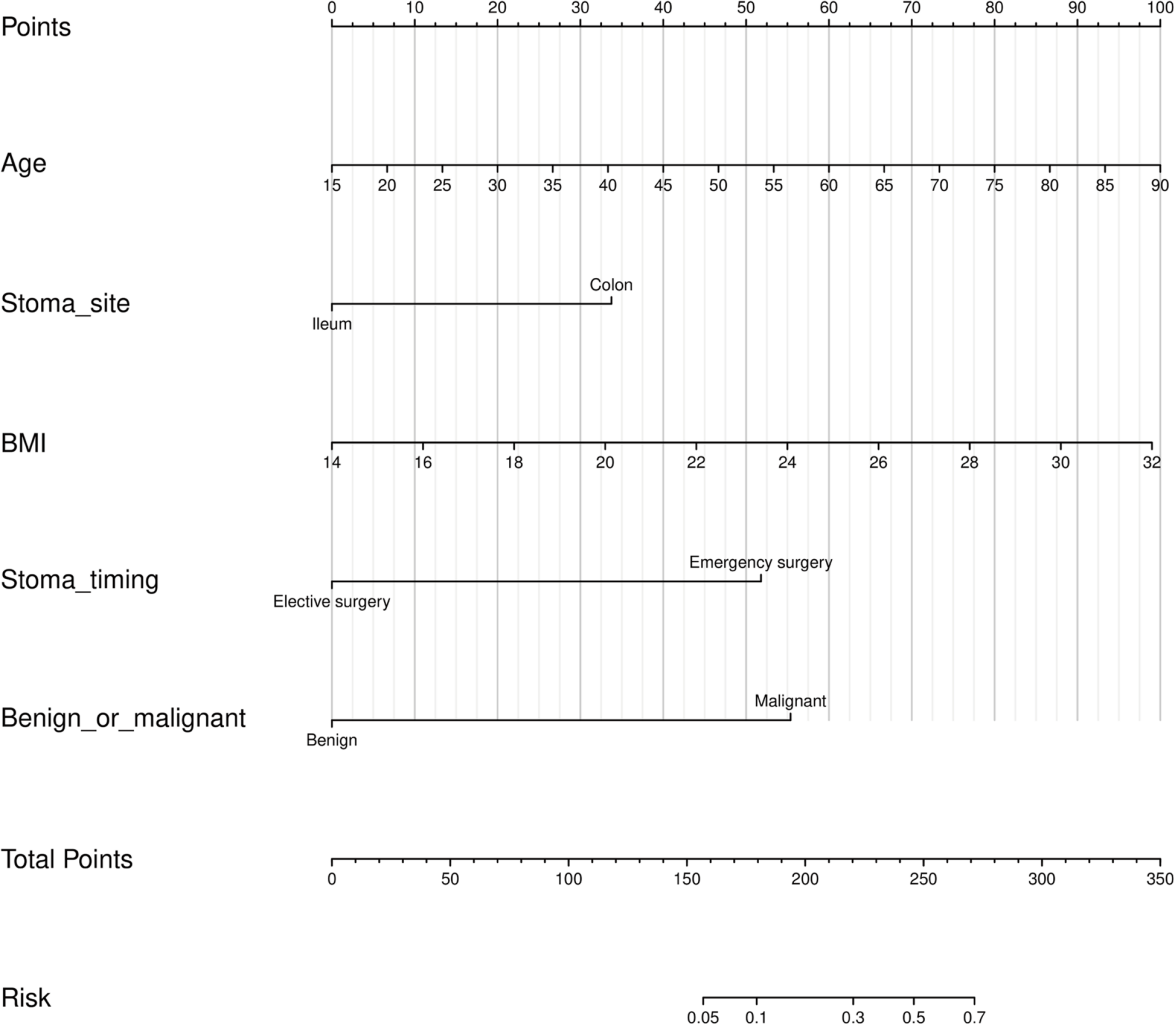
Nomogram to predict the probability of SSIH. The nomogram is used by summing each patient-specific value identified on the scale for each variable. The total points projected on the end of the scales show the risk of SSIH. (SSIH, stoma site incisional hernia)

According to the multivariate analysis, a nomogram clinical prediction model was constructed. The scores of ages and BMI gradually increased with the increase of the value. The score of the stoma site was 33 points, the score of malignant disease was 55 points, and the score of emergency operation was 52 points. Each risk factor was scored individually. Each individual score was added up to get the total score, and the probability corresponding to the total score is the probability of the model predicting the incidence of SSIH. (Fig. 3).

### Evaluating the predictive power of a nomogram model

**Fig. 4:**
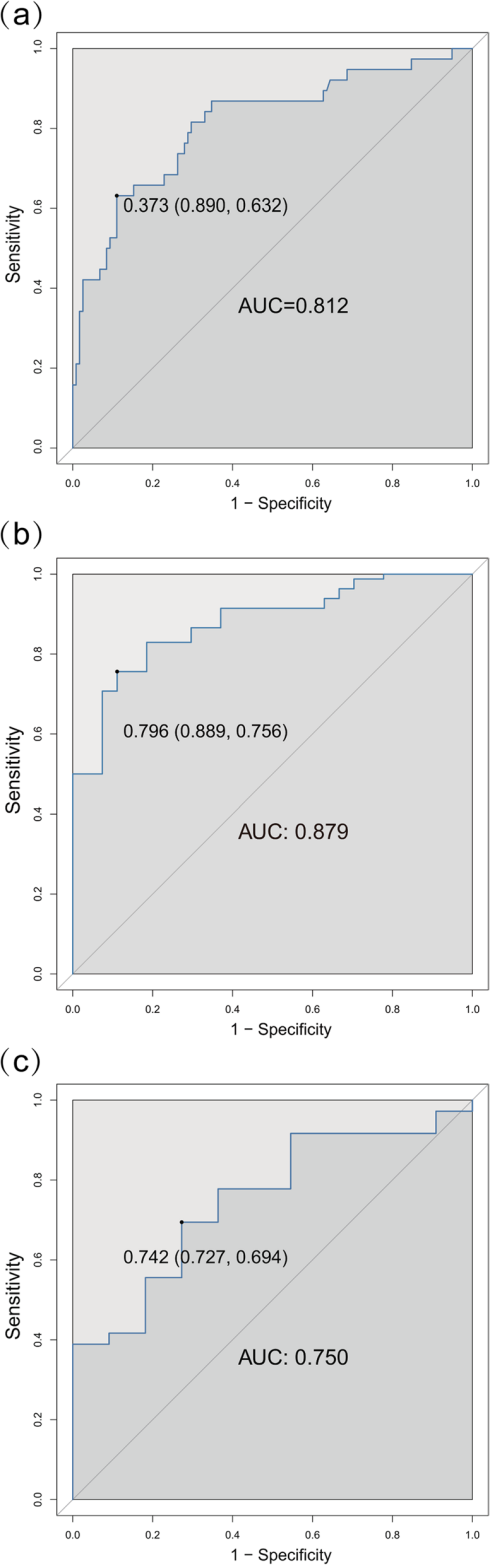
**a** Internally verified ROC curve; (**b**) and (**c**): cross validation ROC curve, (**b**) training cohort (109 cases), (**c**) validation cohort (47 cases)

**Fig. 5:**
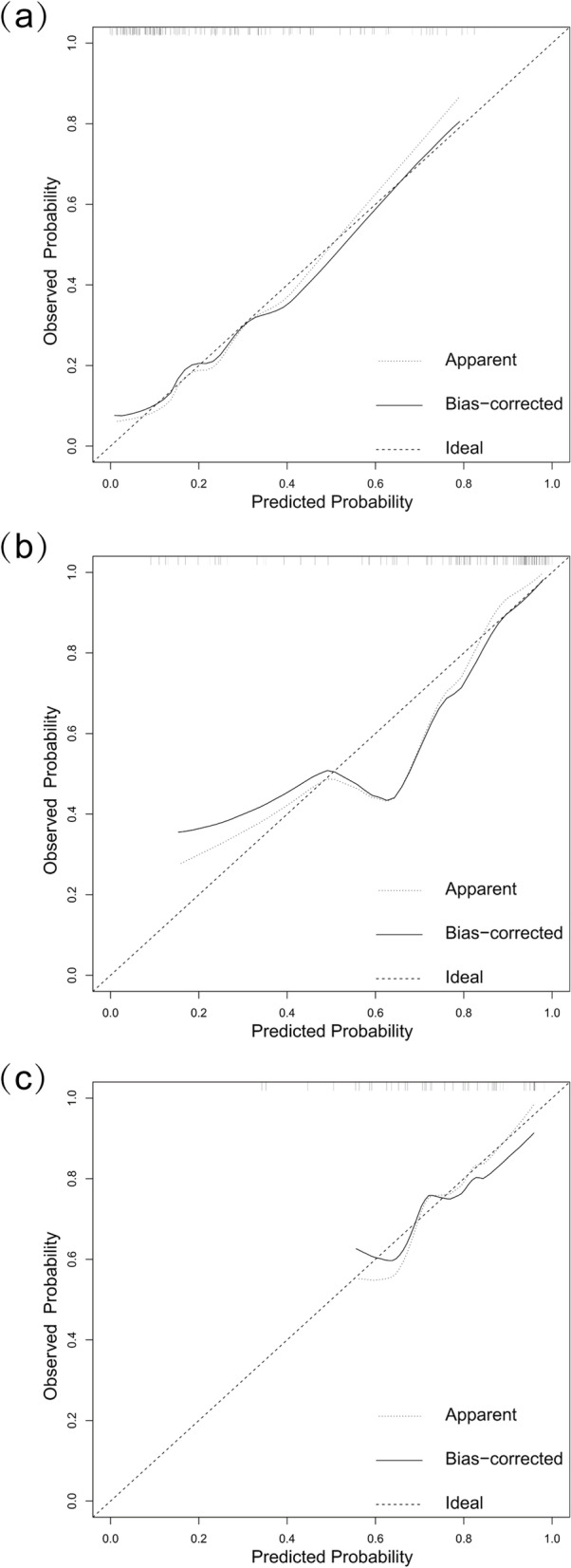
Calibration plots of the nomogram, which is applied to predict the probability of SSIH occurrence in entire cohort (**a**), training cohort (**b**), and verification cohort (**c**)

By drawing the ROC curve, the predictive ability of the nomogram model was evaluated. The results showed that the AUC value of the nomogram prediction model was 0.812 (95% CI: 0.632 ~ 0.890), (Fig. 4a). Besides, after the construction of nomogram, the bootstrap self-sampling technique was performed on the nomogram prediction model for internal verification. After repeated sampling internal verification, the C-index was 0.783. The calibration curve showed that the predicted results of the nomogram model had good consistency with the actual results (Fig. 5a).

Subsequently, 156 patients were randomly divided into the training cohort (109 cases) and the validation cohort (47 cases) according to a 7:3 ratio. The line graph model was applied to the training cohort. The internal verification of the line graph model showed that the area under the ROC curve was 0.879 (Fig. 4b, Table 4). Then the data from the verification cohort was applied to the cross-validation of the line graph model, and the C index was 0.750, indicating that the model had good prediction ability (Fig. 4c, Table 4). The calibration curve results show that no matter the training cohort or the verification cohort, the predicted results of the line graph model have a good consistency with the actual observation results (Fig. 5b and c), and the p values of Hosmer–Lemeshow test are > 0.05 (training cohort *P* = 0.3588, verification cohort *P* = 0.3427).

**Table 4:** Diagnostic efficacy of the nomogram model for estimating the risk of SSIH

Variables	Value	
	**Training Cohort (*n* = 109)**	**Validation Cohort (*n* = 47)**
AUC (95%CI)	0.879 (0.8112-0.9458)	0.750 (0.5946-0.9054)
Threshold (%)	79.59	74.20
Specificity (%)	88.89	72.73
Sensitivity (%)	75.61	69.44
Accuracy (%)	78.90	70.21

## Discussion

The main population of prophylactic enterostomy is elderly patients with colorectal cancer or people with inflammatory bowel disease. There are also some patients in urgent situations like intestinal obstruction, intussusception, intestinal perforation. These patients cannot receive anastomosis after primary resection [9]. Hence, they all need to undergo enterostomy closure in the follow-up, and SSIH often occurs. Some scholars believe that, as long as SSIH occurs for a long enough time, surgery is eventually required [10]. Severe SSIH even requires multiple surgeries, which greatly increases the patient’s economic burden and mental suffering. Currently, the incidence of SSIH varies among different studies, which may be closely related to follow-up time and disease type [11, 12]. Tilney et al. [13] reported that the probability of recurrent incisional hernia after ileostomy was 3.7%, and the probability of recurrent incisional hernia after colostomy was 14.6%. Other related reports pointed out [14, 15] that at least 30% of SSIH occurred within 2 years, and their discomfort symptoms would gradually worsen, and eventually more than half of the patients needed to undergo surgery, and the risk is significantly higher than other patients with abdominal incisional hernia. Therefore, it is particularly important to truly understand the incidence of SSIH and its risk factors. In this study, the follow-up of 156 enterostomy closure patients showed that the incidence of SSIH was 24.4% (38/156), of which the incidence of ileostomy SSIH was 13.1% (8/61), and the incidence of colostomy SSIH was 31.6% (30/95), of which 14 cases underwent hernia mesh repair and recovered well. Other patients are temporarily treated conservatively. Stephen et al. [16] found that the incidence of SSIH was 19% in 365 patients, and the proportion of receiving surgery was 64%, which may be related to the total number of cases and the different types of diseases. They also found that more than one year after ostomy closure is the peak period of SSIH incidence, the median time to onset of SSIH is 32 months after ostomy closure, and SSIH is a late complication of ostomy closure. Therefore, we need to further extend the follow-up time, increase the total number of cases, and enrich the types of diseases.

Related studies have shown that risk factors for abdominal incisional hernia include obesity, hypertension, diabetes, age, emergency surgery, incision infection, etc., and the incidence of SSIH at the stoma site is higher than in the surgery area at other abdominal surgeries, which may be related to the further impact of the stoma itself on local abdominal wall healing [17–20]. Other reports have pointed out that high BMI, loop colostomy and end colostomy are important high-risk factors for the occurrence of SSIH [4, 5, 16]. Obesity may lead to fat accumulation in mesentery and high tension of fascia and muscle tissue, which is more likely to cause poor incision healing and the formation of SSIH. Colostomy results in greater local contamination and larger defects, which further leads to poorer abdominal wall healing and an increased risk of SSIH. This study suggests that colostomy and BMI ≥ 25 kg/m^2^ are independent risk factors for SSIH, which is consistent with literature reports. Stoma method (loop stoma or end stoma) is not risk factor for SSIH in our study. Considering that the risk factors of end stoma and loop stoma for SSIH may be different, our study has subgroup analysis of loop stoma and end stoma. The result shows that malignant tumor is still a high risk factor for end stoma to SSIH, age and stoma timing (emergency/elective surgery) are still high risk factors for loop type stoma to SSIH. As for the other SSIH-related risk factors, none of them have statistical significance in the subgroup analysis, which may be caused by the relatively small sample size, and we hope to increase the number of end stoma and loop stoma cases in future research. In addition, we also found that emergency enterostomy is more prone to result in SSIH, which might be closely related to tissue edema, poor bowel preparation, heavier intraoperative contamination, and more difficult intraoperative operation under emergency conditions. If possible, elective enterostomy should be performed as soon as possible.

Amelung et al. [3] found that stoma prolapse and parastomal hernia are independent risk factors for SSIH, which might be related to the local fascia defect and abdominal wall weakness, and hypertension might lead to poor wound healing through changes in micro-vessels, thereby causing the occurrence of SSIH and becoming one of its risk factors. However, univariate analysis in this study shows no statistical difference between hypertension, diabetes and the occurrence of SSIH, which indicates that hypertension and diabetes might not be the main factors affecting SSIH, prompting us to consider the key risk factors for SSIH. In addition, because the enterostomy patients with parastomal hernia in our hospital were treated with enterostomy closure simultaneous combined with parastomal hernia mesh repair, which strengthened the abdominal wall and prevented the SSIH to an extent, parastomal hernias were not included in this study to avoid bias. Moreover, studies support that malignant tumors and incision infection are considered independent risk factors for SSIH [21, 22]. The results of this study also suggest that malignant tumor is an independent risk factor for SSIH, which might correlate to the tumor's relatively complex disease, the high difficulty of surgical operation, and the changes in the systemic microenvironment caused by the tumor. In the univariate analysis, the difference between incision infection and the occurrence of SSIH was statistically significant, but it was not an independent risk factor for SSIH, which might be related to the bias of the population distribution in this study. Therefore, it is necessary to further increase the overall sample size and reduce bias.

This study also pointed out that age ≥ 68 years can increase the probability of SSIH. As an independent risk factor, it may be related to the weakening of the fascia and the closure of muscle content in the body with the increase of age and pathophysiological changes, which is consistent with others research [16].

The existence of a weak area in the abdominal wall and the elevated intra-abdominal pressure (IAP) are two necessary conditions for the occurrence of hernia. Therefore, we further involved the history of hernia repair, smoking, constipation, COPD, constipation and corticosteroids in the study, which could also lead to the abdominal weakness or high IAP. We found that none of them were associated with the occurrence of SSIH. A previous study has shown that corticosteroids use is a high risk factor for incisional hernia after liver transplantation [23]. This may be related to the dosage of corticosteroids, which needs further investigation.

Currently, many studies have shown that preoperative systemic nutritional status (OPNI, hemoglobin, etc.) and inflammatory markers (lymphocytes, white blood cells, CRP, PLR, NLR, etc.) of patients are closely related to the incidence of complications and even long-term prognosis after gastrointestinal surgery [24–27]. Previous study has s shown that the postoperative systemic state of patients is correlated with incisional hernia [28]. Therefore, in order to comprehensively evaluate the risk factors of SSIH, we included preoperative and postoperative blood test indicators which can reflect patients' systemic status, aiming to discover meaningful findings. We selected pre-WBC, pre-N, pre-LY, pre-Hb, pre-CRP, post-WBC, post-N, post-LY, post-Hb, post-CRP, nutritional indicators (pre-OPNI, post-OPNI) and systemic inflammatory response indicators (pre-NLR, pre-PLR, post-NLR, post-PLR) into the risk correlation study. It was found that none of the above indicators were independent risk factors for the occurrence of SSIH. Our results indicated that nutrition status and inflammatory markers might not be the main risk factor for SSIH.

The technical difficulty and economic burden of surgical treatment of SSIH are significantly higher than other patients with abdominal incisional hernia. Therefore, the prevention of SSIH is particularly important. A multi-center prospective randomized controlled trial pointed out that the addition of prophylactic bio-mesh placement during ostomy closure surgery will not increase surgical complications, and it could effectively prevent the occurrence of SSIH within 2 years follow-up [29], and studies in the Netherlands (NCT03750942) and France (NCT02576184) are currently investigating the role of synthetic patches in these processes. In patients with ileostomy, bio-mesh is more beneficial, which might be related to the underlying disease (ileostomy is more common in inflammatory bowel disease), and the age of the patient (ileostomy patients are relatively young) or other factors (colostomy is more common in elderly patients with malignant tumors), remaining to be further confirmed in their follow-up reports [29].

Understanding the molecular biological processes associated with incisional hernia will provide new therapeutic strategies for the occurrence and prevention of incisional hernia. The current view is that abnormal fibroblast proliferation is the most important cause of incisional hernia [30, 31]. On the other hand, TGF-β1, CTGF, LOX and HIF-1α signaling pathways play important roles in incision healing response, while HMGB1 plays different roles in incision healing and progression. Therefore, TGF-β1:HMGB1 ratio determines the role of HMGB1 in incisional hernia tissues [32–34]. Based on the above studies, we believe that TGF-β1:HMGB1 ratio and myofibroblast proliferation may be the targets for the development and treatment of incisional hernia. Of course, there is still a large gap as to whether the basic biological knowledge of incisional hernia pathogenesis is also applicable to the occurrence of SSIH. Screening of the cellular and molecular mediators associated with fibroblast phenotypic changes in these patient tissues can reveal the relevant molecular biological processes. This is also the basic mechanism research direction that we need to further improve in the future.

As for hypertension, diabetes, incision infection, corticosteroids and other factors reported in previous studies that might be related to the occurrence of SSIH, our study did not reach corresponding conclusions. We hope to further increase the total number of cases and confirm our conclusions in a high-quality multicenter prospective randomized controlled trial.

## Conclusion

The Nomogram constructed in this retrospective study has obtained good results after testing and internal verification, showing that this model has good predictive value for SSIH, and can be applied to clinical patients undergoing ostomy closure surgery for preoperative evaluation and postoperative follow-up guidance. According to this model, preoperative scores were performed on the patients undergoing enterostomy closure to predict the occurrence of SSIH. For patients with high risk of SSIH, surgeons should conduct operation more carefully during the surgery. Besides, according to findings of high-quality clinical research, enterostomy closure combined with prophylactic bio-mesh placement can be a possible solution to significantly reduce the incidence of SSIH. Therefore, combined with the prediction model constructed in this study, the high-risk groups of SSIH after stoma closure can be screened. For these patients, stoma closure combined with prophylactic bio-mesh placement can be the preferred clinical solution.

## Data Availability

All data included in this study are available upon request by contact with the corresponding author.
